# Novel infection by *Mucor hiemalis* kills *Caenorhabditis* hosts through intestinal perforation

**DOI:** 10.1128/iai.00310-25

**Published:** 2026-04-15

**Authors:** Jay Ni, Spencer Weigand, Jessica N. Sowa

**Affiliations:** 1 Department of Biology, West Chester University of Pennsylvania 8510, West Chester, Pennsylvania, USA

**Keywords:** filamentous fungi, *Caenorhabditis*, host-pathogen interactions

## Abstract

The nematode *Caenorhabditis elegans* has emerged as a popular model system to investigate cell biology and host-pathogen interactions. Presently, *C. elegans* is studied as a natural host of intracellular pathogens such as microsporidia and Orsay virus, along with extracellular bacterial and fungal pathogens. The use of *C. elegans* as a model in host-pathogen research is limited by the number of naturally occurring pathogens that infect this organism. Through a sampling project to identify new pathogens of *C. elegans*, we identified the fungus *Mucor hiemalis* as a pathogen of *Caenorhabditis* species. We observed the fungus in the intestinal lumen of wild-caught *Caenorhabditis briggsae,* and co-culturing the wild-caught species with infection reporter *C. elegans* confirmed infection by *M. hiemalis*. This study characterizes the fungal infection by *M. hiemalis* in *Caenorhabditis* nematodes. Fluorescence microscopy with fungal staining revealed the life cycle of *M. hiemalis* within multiple *Caenorhabditis* species at varying growth stages. We observed the killing of nematodes by *M. hiemalis* via intestinal perforation and assessed its host range through a series of lifespan assays. We investigated the food preference of *C. elegans* and determined that nematodes show a preference toward food that contains *M. hiemalis* sporangiospores. Last, we evaluated common *C. elegans* transcriptional immune responses and found that *M. hiemalis* induces the expression of genes related to the intracellular pathogen response. Characterization of this fungal infection in *Caenorhabditis* nematodes will provide new insights into the biology of pathogenic fungi and host immune responses.

## INTRODUCTION

Fungi are a major cause of fungal disease and infection in humans, with an annual burden of over 1 billion globally (1). *Aspergillus, Candida, Cryptococcus, Histoplasma,* and *Mucor* species are the main fungal pathogens responsible for most serious fungal diseases (1). Fungi cause superficial, mucosal, and invasive infections in tissues with a wide range of severity (1). Fungal infections in immunocompromised individuals can be deadly and are responsible for over 1.5 million deaths globally per year (2). Fungal disease is a pressing area of concern as incidence increases due to a range of global issues including climate change and antibiotic resistance (1). Fungal infections also further complicate patients with other chronic and immunosuppressive diseases such as HIV/AIDS, tuberculosis, diabetes, asthma, and cancers (2–7). The diverse array of fungal pathogens and their complex relationship with host immune systems highlights a need for further research to understand host-pathogen interactions and identify potential therapeutic targets.

The nematode *Caenorhabditis elegans* is a powerful and genetically tractable invertebrate system to model pathogenic microbes and their relationship with hosts. In the wild, the typical *C. elegans* diet consists of commensal bacteria from decaying organic matter; however, they also encounter a variety of microbes that are pathogenic (8). *C. elegans* is a natural host to a range of pathogens, including bacteria, fungi, microsporidia, and Orsay virus (9–11). Research using *C. elegans* as a model for host-pathogen interactions has resulted in significant discoveries in microbial pathogenesis and conserved host immune system responses (12).

*C. elegans* lacks an adaptive immune system and does not have any known cellular immunity or specialized immune cells (13). *C. elegans* also lacks homologs of most canonical mammalian pathogen recognition receptors that identify specific pathogens and their associated damage. However, *C. elegans* does have innate epithelial cell immunity that responds to the presence of pathogenic microbes and their virulence factors, and several evolutionarily conserved immunity pathways have been characterized in *C. elegans*. The p38 mitogen-activated protein kinase pathway is a signaling cascade that responds to infections by *Staphylococcus aureus* or *P. aeruginosa* to provide defense (14, 15). The conserved *C. elegans* transforming growth factor β and DAF-2/insulin signaling pathways provide regulation of innate immunity signaling responses and the secretion of antimicrobial peptides (16). The conserved G-protein-coupled receptor DCAR-1 in *C. elegans* responds to physical damage of the epidermal tissue in response to some fungal pathogens (17). Furthermore, studies of *C. elegans* and its naturally occurring intracellular pathogens have led to the discovery of an immunity signaling cascade comparable to mammalian type-I interferon responses called the intracellular pathogen response (IPR) (18). The IPR consists of approximately eighty genes that are upregulated in response to intracellular infections caused by microsporidia *N. parisii* and Orsay virus (18).

*C. elegans* has been used to model human fungal infections, including *Candida, Cryptococcus, Histoplasma,* and *Aspergillus* species to advance understanding of fungal pathogenesis and host immune system responses. *Candida albicans* infects the *C. elegans* intestinal tract via ingestion and causes death by hyphal proliferation (19). The PMK-1/p38 MAPK pathway was found to promote resistance to *C. albicans* infection, but the mechanism has yet to be characterized (19). *Cryptococcus neoformans* infection has also been modeled in *C. elegans*. The capsule of *C. neoformans* is toxic to *C. elegans* when ingested. Several fungal virulence factors were identified using *C. elegans* as a model (20). Insulin/insulin-like growth factor 1 and DAF-16 pathways were observed to provide defense against *Cryptococcus* infection in *C. elegans* (21). The killing of *C. elegans* by *Aspergillus fumigatus* has also been used to model fungal pathogenicity, but the *C. elegans* immune response to this fungal pathogen has yet to be discovered (22). *Drechmeria coniospora* is a natural pathogen of *C. elegans* and has been isolated in wild populations. *D. coniospora* is not known to induce human infection, but the infection in *C. elegans* has been characterized (23). *D. coniospora* attaches to the cuticle of nematodes and penetrates the protective layer to cause infection (23, 24). An induction of antimicrobial peptide genes related to the conserved p38-MAPK cascade was observed in response to the epidermal wounding caused by *Drechmeria* (25). Furthermore, Oomycetes are a diverse group of pathogenic microbes that show fungi-like characteristics of filamentous growth and spore production (26). Oomycete infection has also been modeled in *C. elegans. Myzocytiopsis humicola* is an obligate parasite of *C. elegans* that infects nematodes by attaching and penetrating the cuticle of the nematode and growing within the body cavity with filamentous hyphae. The *C. elegans* transcriptional response to *M. humicola* includes the induction of chitinase-like genes, which reduces pathogen attachment at the start of infection (26). The relatively limited number of natural fungal pathogens of *C. elegans* limits host-pathogen research, particularly given the current rise of fungal threats to human health.

We isolated the fungus *Mucor hiemalis* from a wild sample of *Caenorhabditis briggsae* through the Nematode Hunters project, which is designed to discover novel natural pathogens of wild nematodes. While testing for an IPR response with reporter *C. elegans*, we observed that *M. hiemalis* can infect other *Caenorhabditis* species, including *C. elegans*.

*M. hiemalis b*elongs to the class Zygomycetes, order Mucorales, and genus *Mucor* (27). Fungi from the order Mucorales are opportunistic fungal pathogens that are causative agents of mucormycosis. Mucormycosis infections in immunocompromised hosts are often severe, while infections in immunocompetent hosts are rare (28). In a case study of 851 cases, mucormycosis had a reported mortality of 46% (29). Despite advancements in diagnosis and treatment methods, a rise in the incidence of mucormycosis is of increasing concern as underlying risk factors fuel pathogenesis. *M. hiemalis* is not a typical cause of mucormycosis infection due to its low temperature tolerance of 33°C, but at least five cases have been diagnosed to date (27, 30). Three cases of cutaneous *M. hiemalis* infections have been diagnosed: two instances of *M. hiemalis* infection were identified in young immunocompetent girls, and one in a 76-year-old woman (31–33). Subcutaneous infections by *M. hiemalis* have been identified in a 44-year-old immunocompetent patient and an immunodeficient diabetic patient (34, 35). More recently, an increase in *Aspergillus* and *Mucor* infections has been observed in patients with COVID-19, showing a coinfection dynamic between fungi and virus (36, 37). The increase in risk and incidence of fungal diseases like mucormycosis highlights the need for the development of antifungal therapies and understanding of immune mechanisms through research on fungal-host interactions.

We present novel research establishing *C. briggsae* as a natural host of the fungal pathogen *M. hiemalis*. Our results demonstrate that *M. hiemalis* is ingested by *Caenorhabditis* nematodes, where it colonizes the intestine with vegetative growth and causes intestinal perforation and eventual death. We show that *M. hiemalis* infects *Caenorhabditis* nematodes with a wide host range that includes *C. briggsae, C. elegans,* and several more distantly related species. We characterized the pathogenesis of *M. hiemalis*, showing that the fungal sporangiospores are ingested and then germinate into hyphae and chlamydospores within the nematode intestine, causing physical damage to the intestinal barrier. Finally, we assessed gene expression changes for a selection of genes associated with innate immunity mechanisms in *C. elegans* and found that *M. hiemalis* induces expression of a subset of genes associated with the IPR. Our findings characterize a new naturally occurring host-pathogen relationship between *Caenorhabditis* nematodes and *M. hiemalis*, which opens new avenues for studying host immune responses and pathogenesis factors of Mucorales fungi using nematode models.

## RESULTS

### Novel intestinal fungus *Mucor hiemalis* identified in wild *C. briggsae*

**Fig 1:**
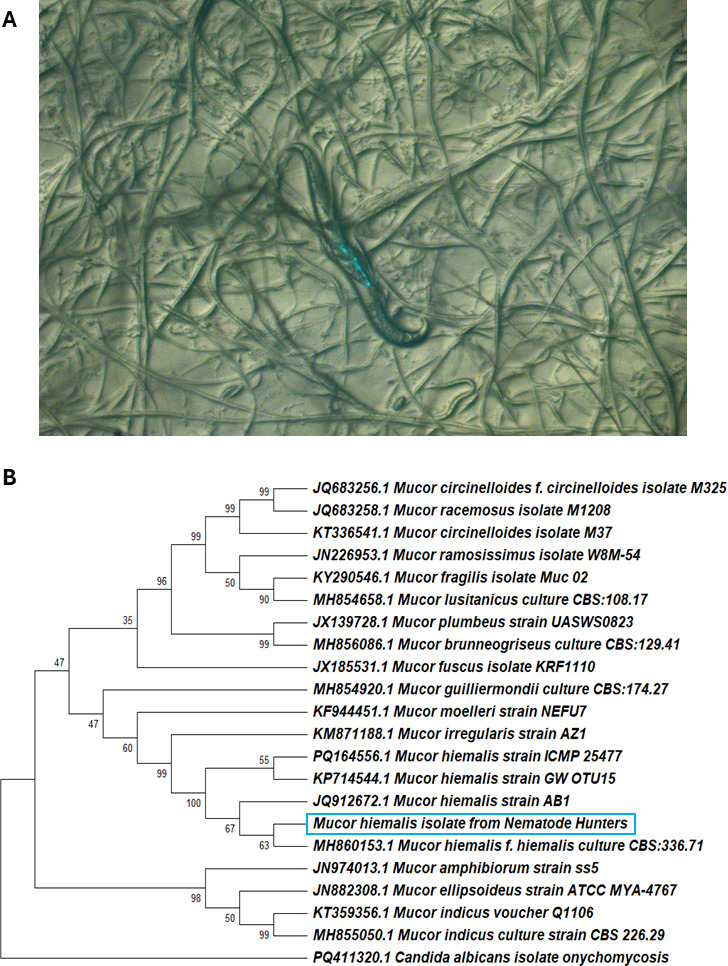
Identification of *M. hiemalis* and *C. briggsae* by ITS sequencing. (**A**) Co-culture experiment performed with *C. briggsae* SOW21 wild isolate and SOW6 *pals-5p::GFP* IPR infection reporter *C. elegans* showing positive *pals-5::GFP* expression. (**B**) Evolutionary relationship of *Mucor* sp. in relation to and similarity to *M. hiemalis* isolated from a leaf litter sample. Mucor sequences were gathered from NCBI BLASTN. Phylogeny was inferred using the maximum likelihood method and the Hasegawa-Kishino-Yano (1985) model of nucleotide substitutions, and the tree with the highest log likelihood (−4,797.06) is shown. The evolutionary rate differences among sites were modeled using a discrete Gamma distribution across four categories (*+G*, parameter = 0.5606). The analytical procedure encompassed 22 coding nucleotide sequences using the 1st, 2nd, 3rd, and non-coding positions with 746 positions in the final data set. Evolutionary analyses were conducted in MEGA12.

We first isolated *Mucor hiemalis* and its host *C. briggsae* from a leaf litter sample from Ridgway, Pennsylvania, USA, on September 30, 2023 (Table 1). Co-culturing the wild nematodes with IPR reporter *C. elegans* resulted in *pals-5p*::GFP expression, which typically indicates infection transmission (Fig. 1A). We observed that nematodes grown on the fungus showed a distended intestinal phenotype after staining with the chitin binding dye Direct Yellow 96 (DY96). Microscopy revealed that the fungus is ingested by nematodes. Vegetative growth of the fungus was observed inside the intestinal lumen of *C. briggsae,* and some fungus colonized the body cavity of dead nematodes. As *C. elegans* infection by other dimorphic and filamentous fungi, including *C. neoformans* and *C. albicans,* was previously studied, we pursued sequencing methods to identify the pathogen. Sanger sequencing and BLASTN search of the internal transcribed spacer 1 (ITS) gene of the wild isolate JNSP1 revealed a high sequence similarity with other *M. hiemalis* isolates. Other ITS sequences of *Mucor* isolates were obtained, and the resulting phylogenetic tree revealed a distinct clade of *M. hiemalis,* including the JNSP1 isolate from Nematode Hunters (Fig. 1B). The sequencing of the wild host nematode cultured with *M. hiemalis* was determined to be *C. briggsae* by small-subunit ribosomal RNA and internal transcribed spacer 2 sequencing.

**TABLE 1:** Nematode Strains used in this study

Strain name	Species	Genotype	Source
N2	*C. elegans*	Wild type (N2)	CGC
SOW6	*C. elegans*	*rde-1* (*ne219*)V; jyIs8[*pals-5p::GFP*; *myo-2p::mCherry*] X	Cross of WM27 and ERT54
SOW21	*C. briggsae*	Wild isolate	Isolated from Ridgway, PA
DF5081	*C. japonica*	Wild isolate	CGC
JU1373	*C. tropicalis*	Wild isolate	CGC
PS312	*P. pacificus*	Wild isolate	CGC

### Ingested *M. hiemalis* sporangiospores germinate hyphal bodies and accumulate inside the *Caenorhabditis* nematode intestine

**Fig 2:**
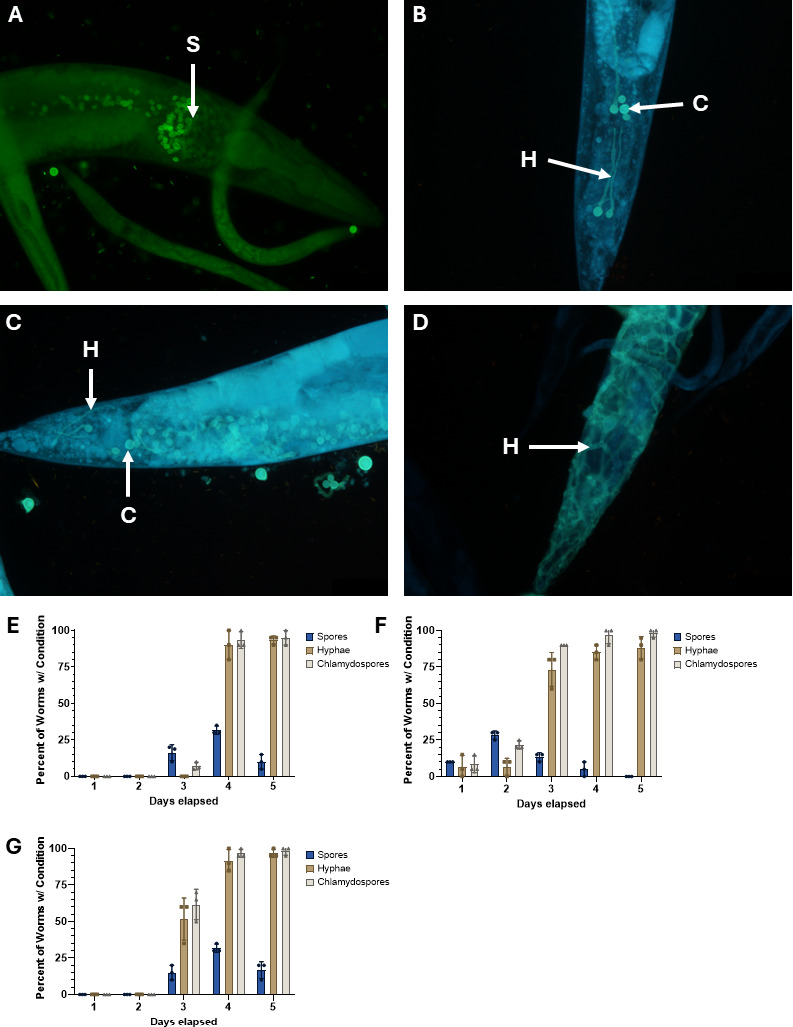
Ingested *M. hiemalis* sporangiospores germinate hyphal bodies and accumulate inside the *Caenorhabditis* nematode intestine. Fluorescence micrographs of *M. hiemalis* growth stages stained with direct yellow 96 in infected wild-type N2 *C. elegans*. Nematodes were scored for fungal growth conditions in biological triplicates. The percentage of *C. elegans* containing *M. hiemalis* sporangiospores (S), hyphae (H), and chlamydospores (C) was quantified. (**A**) *M. hiemalis* sporangiospores are ingested by *Caenorhabditis* nematodes and accumulate in the intestinal lumen. (**B**) Vegetative growth from sporangiospores to filamentous hyphae at the posterior end of the intestine. Chlamydospores arise from hyphal segments during asexual spore production. (**C**) Accumulation of vegetative growth forms in the posterior end of the nematode. (**D**) Hyphal growth breaches the intestinal lumen and enters into the nematode body cavity. (**E**) *M. hiemalis* growth stage counts in N2 *C. elegans* across 5 days of observation by fluorescence microscopy with (**E**) larval stage 4, (**F**) adult day 1, and (**G**) adult day 4 nematodes. Graphs show the average percentage of nematodes showing each fungal stage from three independent experiments combined (*n* = 20 for each experiment). Error bars show standard deviation.

Next, we characterized the life cycle of *M. hiemalis* when ingested by nematode hosts with staining and microscopy methods (Fig. 2). Reproductive stages of *M. hiemalis* were identified within nematode hosts, including sporangiospores (S), chlamydospores (C), and filamentous hyphae (H) (Fig. 2A through D). The infection of *Caenorhabditis* hosts begins with spore ingestion, as sporangiospores fill the intestinal lumen (Fig. 2A). Sporangiospores germinate into hyphae and chlamydospores and accumulate in the posterior end of the nematode (Fig. 2B and C). Later time points of fungal accumulation revealed fungus germinating outside of the intestinal lumen and throughout the body cavity of the nematode (Fig. 2D). Fungal stages were characterized across 5-day time points for a range of nematode growth stages (Fig. 2E through G). When *M. hiemalis* was introduced to larval stage 4 (Fig. 2E) *C. elegan*s, sporangiospores were first observed in the intestine on the third day after the introduction of sporangiospores. On day 1 adult nematodes, sporangiospores were observed on the first day (Fig. 2F). We attributed the difference in sporangiospore ingestion between the L4 nematodes and day 1 adults to nematode age and size differences. *C. elegans* mouth size is about 4 µm at the L4 stage, and it grows larger with nematode age (38). We measured *M. hiemalis* sporangiospores to be ~6 µm in diameter, and the L4 nematodes had grown to a larger adult stage by the third day of observation. The known size of *M. hiemalis* sporangiospores was useful in distinguishing between sporangiospores and chlamydospores. Chlamydospores vary in size but are generally larger and appear more spherical than the ellipsoid shape of sporangiospores. We concluded that nematodes cannot ingest chlamydospores due to size, but rather, the chlamydospores observed within the nematode were a product of sporangiospore germination. Sporangiospore counts decreased after initial ingestion in all stages as sporangiospores germinated into alternative growth forms. A high frequency of nematodes contained hyphae and chlamydospores by the fourth and fifth days of infection (Fig. 2E through G; Fig. S1). A difference in *M. hiemalis* ingestion was observed in adult day 4 nematodes, as no sporangiospores were observed in the intestine until the third day (Fig. 2G). We attributed the reduction of ingestion to reduced pharyngeal pumping due to age-related muscle degeneration and physical accumulation of fungus inside the pharynx (39).

### *Caenorhabditis* nematodes prefer *E. coli* mixed with *M. hiemalis* sporangiospores

**Fig 3:**
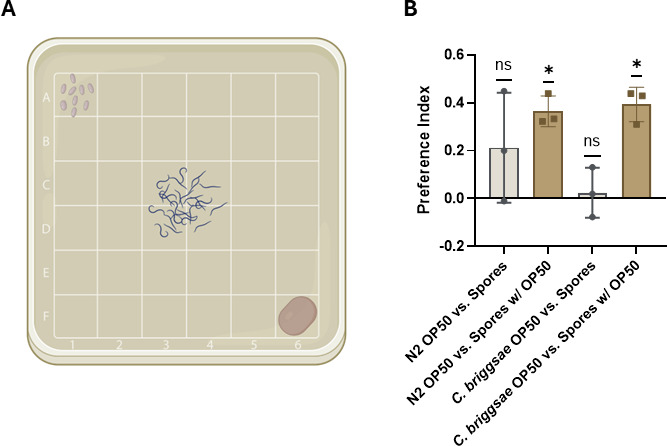
*C. elegans and C. briggsae* show a preference for ingesting *M. hiemalis* sporangiospore and *E. coli* mixture. Preference indices were calculated from triplicate experiments tested in parallel using 100 nematodes per group. A one-sample *t*-test was used to compare the preference score to no preference. (**A**) A schematic representation of an assay plate used for calculating preference indexes. *M. hiemalis* sporangiospores were placed in the top left corner, and OP50-1 *E. coli* was placed in the bottom right corner; 100 *C. elegans or C. briggsae* were introduced in the middle four squares of the plate. (**B**) Food preference index of *M. hiemalis* sporangiospores and sporangiospore mixture compared to OP50-1. *C. elegans* showed no preference between fungal sporangiospores in water compared to OP50–1 (one-sample *t*-test, *P* = 0.2500, *n* = 3). *C. elegans* showed a significant preference toward a fungal mixture of sporangiospores and OP50-1 over OP50-1 alone (one-sample *t*-test, *P* = 0.0102, *n* = 3). *C. briggsae* showed no preference between fungal sporangiospores in water compared to OP50–1 (one-sample *t*-test, *P* = 0.7222, *n* = 3). *C. briggsae* showed a significant preference toward a fungal mixture of sporangiospores and OP50-1 over OP50-1 alone (one-sample *t*-test, *P* = 0.0110, *n* = 3). The experiment was repeated three times independently. Error bars show standard deviation.

Given that we observed nematodes ingesting the fungus, we investigated the preference of *Caenorhabditis* nematodes when presented with *M. hiemalis* or alternative food choices. Nematodes can exhibit a preference toward high-quality food that supports growth and avoidance of foods that do not support growth (8). Nematodes have also demonstrated pathogen avoidance after initial ingestion by olfactory learning with *P. aeruginosa* (40) and have demonstrated lawn-leaving behavior with the pathogenic bacteria *Serratia marcescens* by chemosensation (41). We assayed if *C. elegans* displayed behavioral preference toward *M. hiemalis* through a choice assay (Fig. 3A). Preference indices were calculated and analyzed with a one-sample *t*-test to a theoretical mean of 0, where 0 indicates no preference. *C. elegans* did not show a significant preference toward OP50-1 *E. coli* compared to *M. hiemalis* sporangiospores alone (Fig. 3B). *C. elegans* showed a preference to the mixture of *M. hiemalis* and OP50-1 compared to *M. hiemalis* or OP50-1 alone (*P* = 0.0102, one-sample *t*-test). Similarly, we assayed the food preference of the nematode species originally inoculated with *M. hiemalis* and found that *C. briggsae* also does not show a significant preference toward OP50-1 *E. coli* compared to *M. hiemalis* sporangiospores alone (Fig. 3B). *C. briggsae* also showed a preference to the mixture of *M. hiemalis* and OP50-1 compared to *M. hiemalis* or OP50-1 alone (*P* = 0.0110, one-sample *t*-test). Interestingly, the mixture of fungal and bacterial components is more comparable to nematode food sources in natural ecosystems than isolated bacterial strains. The sporangiospore mixture with OP50-1 was utilized for infection protocols in future experiments.

### *M. hiemalis* kills *Caenorhabditis* nematodes with a wide host range

**Fig 4:**
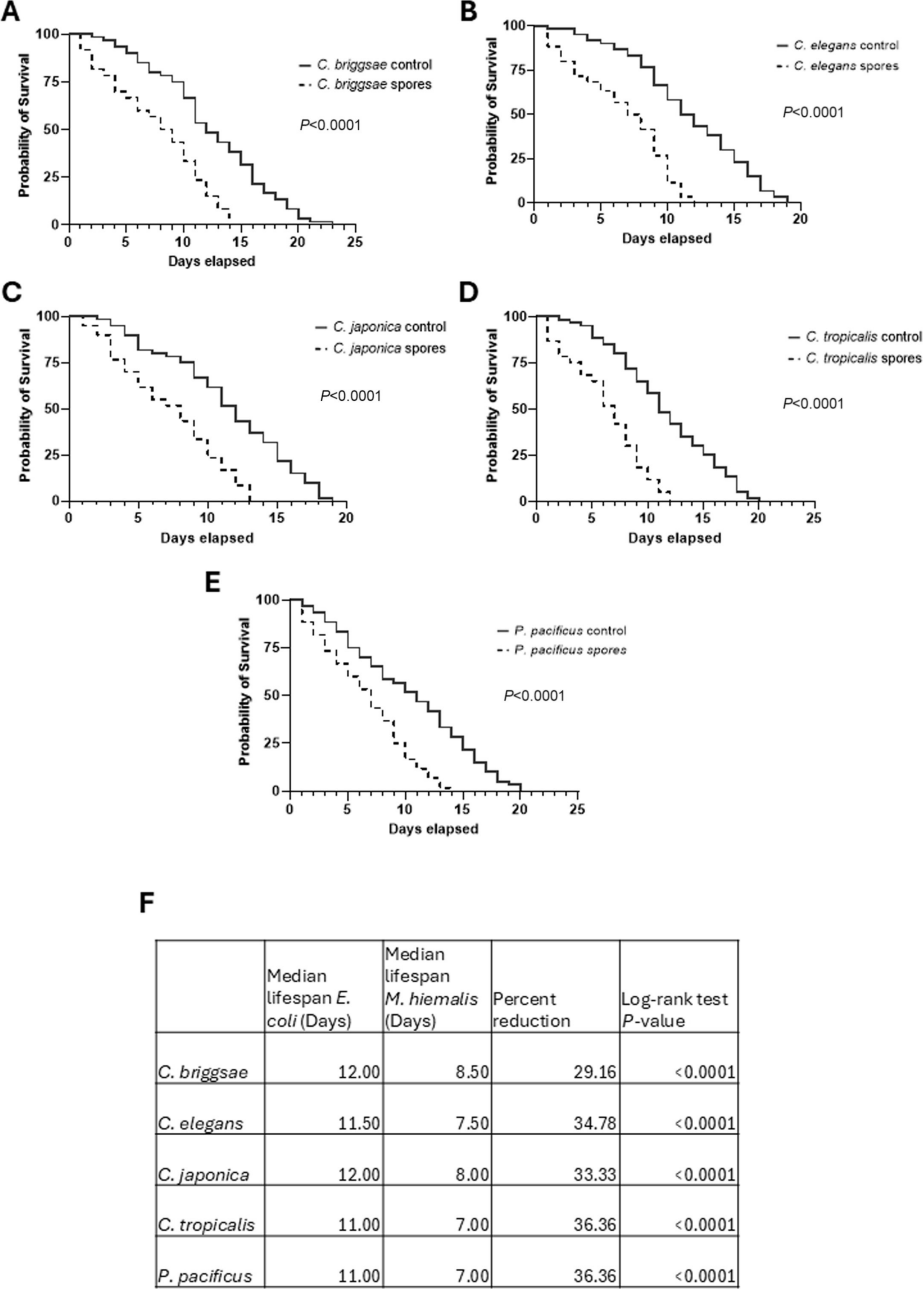
*M. hiemalis* kills *Caenorhabditis* nematodes with a wide host range. Survival assays of (**A**) *C. briggsae* (SOW21), (**B**) *C. elegans* (N2), (**C**) *C. japonica* (DF5081), (**D**) *C. tropicalis* (JU1373), and (**E**) *P. pacificus* (PS312) grown on *M. hiemalis* + OP50 infection plates or OP50 control plates. Infection by *M. hiemalis* significantly reduced lifespan in each species tested (log-rank test, *P* < 0.001). Graphs show combined lifespan from three independent biological replicates (*n* = 20 for each experiment). (**F**) Table showing median lifespan and percent reduction with Log-rank (Mantel-Cox) tests comparing lifespans of nematodes grown with vs. without *M. hiemalis*.

After confirming *M. hiemalis* ingestion in nematodes, we assayed the survivability of nematodes to observe if *M. hiemalis* infection reduced their lifespan. We performed lifespan analysis on a range of species, including our original wild isolate of *C. briggsae* (Fig. 4A), *C. elegans* (Fig. 4B), *C. japonica* (Fig. 4C), and *C. tropicalis* (Fig. 4D). These species are relatively distantly related, sharing a common ancestor more than 80 mya (42). We also assayed the survivability of a nematode species outside of the rhabditids group, *Pristionchus pacificus* (Fig. 4E). *M. hiemalis* significantly reduced the survivability of all species tested with similar percent reductions in median survival (Fig. 4F). *C. briggsae* on infection plates lived to a median survival of 8.50 days compared to 12 days on control OP50 plates. *C. elegans* on infection plates lived to a median survival of 7.50 days compared to 11.50 days on control OP50 plates. *C. japonica* on infection plates lived to a median survival of 8.00 days compared to 12.00 days on control OP50 plates. *C. tropicalis* on infection plates lived to a median survival of 7.00 days compared to 11.00 days on control OP50 plates. *P. pacificus* on infection plates lived to a median survival of 7.00 days compared to 11.00 days on control OP50 plates; *M. hiemalis* reduced the survivability of all species tested with similar percent reductions in median survival (*C. briggsae* 29.16%; *C. elegans* 34.78%; *C. japonica* 33.33%; *C. tropicalis* 36.36%; and *P. pacificus* 36.36%). A comparison of survival curves using a Log-rank (Mantel-Cox) test revealed a statistically significant reduction in all species compared to controls (*P* < 0.0001). *M. hiemalis* consistently reduced the lifespan of all four *Caenorhabditis* species and a non-*Caenorhabditis* species, suggesting that it has a wide host range and consistent pathogenesis.

### *M. hiemalis* infection kills nematodes by perforating the intestinal barrier

**Fig 5:**
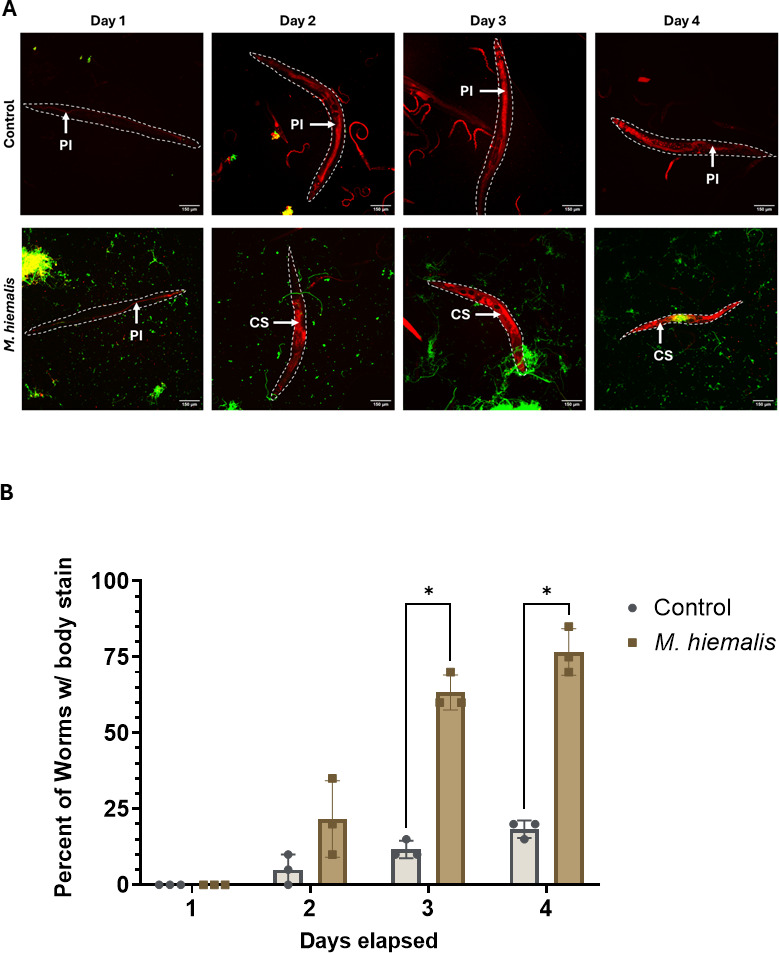
*M. hiemalis* infection perforates the intestinal barrier. (**A**) Fluorescence micrographs of N2 *C. elegans* grown on *M. hiemalis* infection plates and OP50 control plates after DY96 (green) and PI (red) staining. PI staining outside of the intestinal lumen indicates that the intestinal barrier is no longer intact. Images show propidium iodide (PI) staining of intact intestine and propidium iodide body cavity staining (CS) with perforated intestine. (**B**) Percentages of infected N2 nematodes and controls with PI staining in the body cavity. Nematodes were scored positive for PI staining if fungus was present, and PI staining occurred outside of the intestine. Nematodes grown on *M. hiemalis* infection plates showed a higher percentage of the population with PI staining than nematodes grown on control plates (unpaired *t*-test, * indicates *P* < 0.001). The graph is the average of three independent experiments (*n* = 20). Error bars show standard deviation.

To better understand the mechanism by which *M. hiemalis* kills nematodes, we examined the intestinal barrier integrity relative to fungal growth. The previous microscopy experiment revealed *M. hiemalis* growth outside of the intestinal lumen at later time points; hence, we hypothesized that *M. hiemalis* growth perforates the intestine. To examine the intestinal barrier integrity, we fed nematodes with propidium iodide (PI) stain. This stain remains contained within the intestinal lumen of intact intestines but will leak into the body cavity in nematodes with a perforated intestine (Fig. 5A). Nematodes were scored positive for intestinal perforation if the body cavity was stained (Fig. 5B). No PI body cavity staining occurred on the first day in either control or infection plates. On the second day, 5% of control nematodes showed body cavity staining by PI compared to 22% of infected nematodes. PI staining occurred in control nematodes, likely due to cellular degeneration associated with increasing age. Nematodes infected with *M. hiemalis* had significantly more nematodes with PI staining of the cavity compared to controls on the third (*P* = 0.0002, unpaired *t*-test) and fourth day (*P* = 0.0002, unpaired *t*-test). As we observed PI staining in infected nematodes, we concluded that the accumulation of *M. hiemalis* in the intestine induces perforation.

### *M. hiemalis* infection induces intracellular pathogen response genes in *C. elegans*

**Fig 6:**
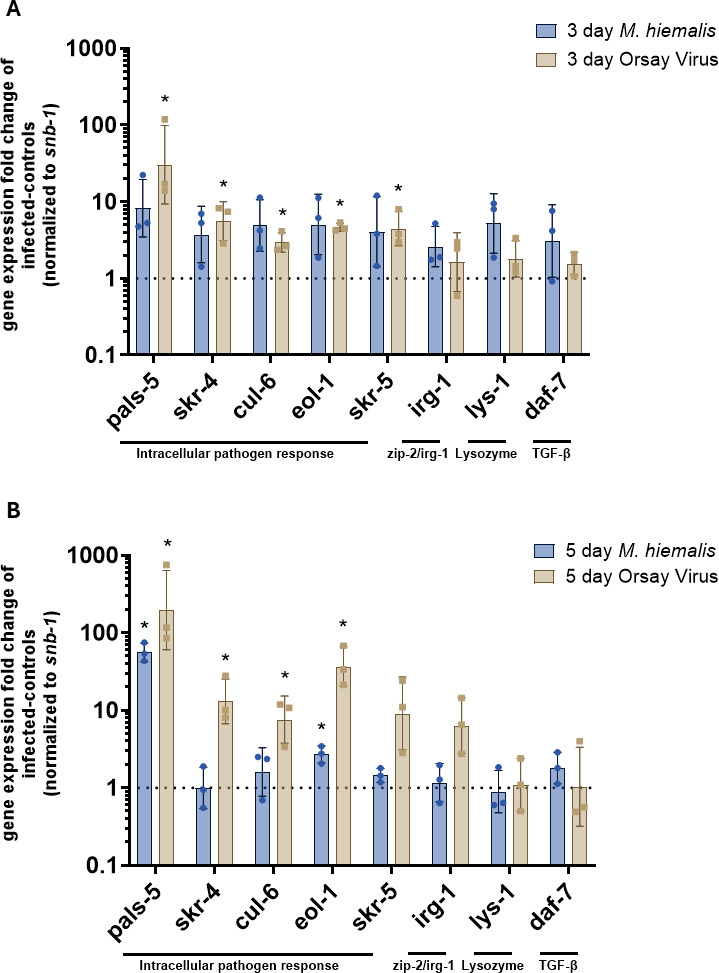
*M. hiemalis* infection induces intracellular pathogen response genes in *C. elegans*. 1,000 synchronized adult day 1 N2 *C. elegans* were incubated on fungal infection plates, Orsay virus plates, and OP50-1 control plates. Nematodes were collected at 3- or 5-day time points after infection. The experiment was repeated three times independently. Relative fold changes in gene expression were normalized to *snb-1* and compared to uninfected control groups. (**A**) Gene expression fold change of seven IPR and innate immunity genes in *M. hiemalis* or Orsay virus-infected nematodes after 3 days. Error bars show standard deviation. Significance was determined by comparing gene expression fold changes of infected vs. controls to a value of 1 with one-sample *t*-tests, * indicates *P* < 0.05 IPR genes *pals-5* (*P* = 0.0376), *skr-4* (*P* = 0.0362), *cul-6* (*P* = 0.0234), *eol-1* (*P* = 0.0021), and *skr-5* (*P* = 0.0374) were significantly upregulated in Orsay virus-positive control nematodes. No genes were significantly upregulated in *M. hiemalis*-infected nematodes. (**B**) Gene expression fold change of seven IPR and innate immunity genes of *M. hiemalis* and Orsay virus-infected nematodes after 5 days. Error bars show standard deviation. Significance was determined by comparing gene expression fold changes of infected vs. controls to a value of 1 with one-sample *t*-tests, * indicates *P* < 0.05 IPR genes *pals-5* (*P* = 0.0162), *skr-4* (*P* = 0.0217), *cul-6* (*P* = 0.0377), and *eol-1* (*P* = 0.0087) were significantly upregulated in Orsay virus-positive control nematodes. IPR genes *pals-5* (*P* = 0.0016) and *eol-1* (*P* = 0.0217) were significantly upregulated in *M. hiemalis*-infected nematodes.

Next, we investigated common nematode immune responses to determine if any were induced by *M. hiemalis* infection. Since *C. elegans* lacks adaptive immunity, these nematodes rely on innate immune mechanisms to protect and clear infections (13). The *C. elegans* intracellular pathogen response (IPR) plays an important role in providing intestinal immunity against Orsay virus and the microsporidia *N. parisii* (18, 43). *C. elegans* also has an established p38 MAP kinase pathway that contributes to innate immunity (15) and shows antimicrobial peptide expression in the epidermis in response to *D. coniospora* infection through regulation of the TGF-β signaling pathway (16). Last, *C. elegans* can respond to pathogen infection by inducing the ZIP-2/IRG-1 pathway to respond to toxins (44). We assessed activation of common *C. elegans* immune responses during *M. hiemalis* infection using a collection of IPR genes and other innate immunity genes associated with p38 MAPK, TGF-β, and zip-2/irg-1 pathways (Fig. 6; Fig. S2).

ΔΔCt gene expression analysis showed that the majority of the IPR and innate immunity genes in *C. elegans* first infected with *M. hiemalis* at larval stage 4 or adult day 2 stages were not significantly induced (Fig. S2A). A one-sample *t*-test was performed to compare gene expression fold change to a theoretical mean of 1, indicating no change. In nematodes infected at larval stage 4, the gene expression of *irg-1* was significantly decreased (*P* = 0.0305). The reduction of *irg-1* was unexpected, as previous studies found that *irg-1* is a transcriptional response that is upregulated in the presence of Exotoxin A, which is secreted by the bacterial pathogen *P. aeruginosa*. The cause for the reduction of *irg-1* in young nematodes is unknown. In nematodes infected at adult day 2, *pals-5* showed a nearly significant increase (*P* = 0.0518), and *eol-1* showed a nearly significant reduction (*P* = 0.0514). The other innate immunity genes were not significantly changed.

Following the results of the first analysis of the IPR and other immunity genes in *C. elegans* in response to *M. hiemalis* infection, we observed the response at later time points for nematodes infected at adult day 1 and collected at 3 or 5 days post-infection. Nematodes were also infected with Orsay virus in parallel to *M. hiemalis* infection to serve as a positive control for IPR gene expression. We also cultured nematodes with Exotoxin A expressing *E. coli* to serve as a positive control for *irg-1* expression following the observed downregulation of *irg-1* results in the first analysis (Fig. S2B). A one-sample *t*-test was performed to compare gene expression fold change to a theoretical mean of 1, indicating no change. In nematodes cultured with *M. hiemalis* for 3 days (Fig. 6A), we did not observe an upregulation in any IPR or immunity genes; however, *pals-5* showed a nearly significant upregulation (*P* = 0.0515). As expected, the gene expression of *C. elegans* cultured with Orsay virus for 3 days showed a significant upregulation in all IPR genes tested (*P*<0.05, Fig. 6A). The remaining immunity genes (*irg-1, lys-1,* and *daf-7*) were not significantly upregulated in response to Orsay virus infection after 3 days. The nematodes cultured with ToxA-secreting *E. coli* for 3 days (Fig. S2B) showed the expected results of an upregulation of *irg-1* (*P* = 0.0033).

Nematodes cultured with *M. hiemalis* for 5 days (Fig. 6B) showed an upregulation of *pals-5* (*P* = 0.0016) and *eol-1* (*P* = 0.0217). The nematodes cultured with Orsay virus for 5 days showed a significant upregulation in all the tested IPR genes except for *skr-5*. IPR genes *pals-5* (*P* = 0.0162), *skr-4* (*P* = 0.0217), *cul-6* (*P* = 0.0377), and *eol-1* (*P* = 0.0087) were significantly upregulated, and the remaining immunity genes (*skr-5, irg-1, lys-1,* and *daf-7*) were not significantly upregulated in response to Orsay virus infection after 5 days. The nematodes cultured with ToxA-secreting *E. coli* for 5 days (Fig. S2C) showed the expected results of an upregulation of *irg-1* (*P* = 0.0211). The gene expression analysis of *C. elegans* IPR and immunity genes revealed an upregulation in two IPR genes at the 5-day time point, which suggests a novel transcriptional response to fungal infections.

## DISCUSSION

The study of host-pathogen interactions using the *C. elegans* model is limited by the number and variety of naturally occurring nematode pathogens that have been identified. Here, we identified the human-pathogenic dimorphic fungus *M. hiemalis* as a natural fungal pathogen of *Caenorhabditis* nematodes and developed a system to model fungal pathogenicity. We characterized the life cycle of *M. hiemalis* in nematode hosts and found that infection begins with spore ingestion and vegetative growth accumulates in the posterior end of the nematode. Nematodes showed a preference toward a mixture of *M. hiemalis* sporangiospores and OP50-1. We observed a significant reduction in survivability in multiple nematode species and determined that *M. hiemalis* kills nematodes by perforating the intestine. We observed significant induction of two IPR genes in response to the fungal infection. In total, we have characterized a novel fungal pathogen of *Caenorhabditis* nematodes and provided insights into their host-pathogen relationship.

To investigate the feeding preferences of *Caenorhabditis* hosts, we designed an assay to determine whether *C. elegans* and *C. briggsae* preferred *M. hiemalis* or a standard laboratory food source, OP50-1 *E. coli*. Our observations revealed a significant preference for a mixture of *M. hiemalis* sporangiospores and OP50-1 compared to OP50-1 alone, which we subsequently used in further experiments. Interestingly, both species did not show a significant preference for *M. hiemalis* alone; however, in both conditions involving the fungus, nematodes showed a positive average preference. Literature shows that *C. elegans* employs chemosensing to detect chemical signals and secondary metabolites from surrounding microbes, and they can distinguish between high- and poor-quality food (8, 45). Our findings raise questions about whether *Caenorhabditis* nematodes are detecting metabolites secreted by *M. hiemalis* and whether nematodes sense that *M. hiemalis* can support growth. While we do not know if *Caenorhabditis* nematodes derive nutritional value from ingesting *M. hiemalis* sporangiospores and fungal structures, it is possible that they are attracted to fungal nutrients or fermentation byproducts. Furthermore, *M. hiemalis* might secrete chemicals that mimic other processes that are attractive to nematodes. For instance, the nematode-trapping fungus *Arthrobotrys oligospora* attracts nematodes by secreting compounds that mimic sex and food cues (46). Olfactory mimicry could potentially play a role in the initial attraction of nematodes to *M. hiemalis*.

Given that specific behaviors are linked to the initial attraction of *C. elegans and C. briggsae* to food sources, it is plausible that these nematodes could also develop learned avoidance towards *M. hiemalis* following initial ingestion. *C. elegans* have been shown to avoid certain pathogenic bacteria, such as *S. marcescens* and *P. aeruginosa,* after an initial encounter. When fed *S. marcescens*, *C. elegans* recognizes serrawetin, a chemical secreted by the bacterium, and subsequent detection by G protein and toll-like pathways is responsible for lawn-leaving behavior (41). A similar interaction is observed with *P. aeruginosa,* where *C. elegans* exhibits transgenerational avoidance behaviors by recognizing bacterial small RNAs (47). Our current study only captured the initial attraction of *M. hiemalis* due to the use of sodium azide to immobilize the nematodes after their first choice. Future investigations could reveal whether nematodes also develop learned avoidance behaviors toward *M. hiemalis*.

Our first analysis of larval 4 and adult day 2 *C. elegans* transcriptional immune response to *M. hiemalis* did not reveal a significant upregulation of common innate immunity genes typically associated with viral, bacterial, and fungal pathogens of *C. elegans*. Although we observed a near-significant upregulation of *pals-5* (*P* = 0.0518) in adult day 2 nematodes, the level of *pals-5* expression detected by qPCR was lower than the anticipated levels based on our co-culture assays. Several factors could account for this discrepancy. In co-culture tests, *pals-5p::GFP* expression typically became apparent after 3 days post-infection. The qPCR experiment to quantify gene expression was conducted after only 2 days of infection. We hypothesized that extending the qPCR analysis to time points further than 3 days post-infection might reveal higher *pals-5* expression levels that agreed with the previous co-culture results. Additionally, normalization of total RNA with *snb-1* could be diluted by the presence of excess progeny in our samples that could not be infected by the *M. hiemalis* due to their size.

The second analysis of the *C. elegans* immune transcriptional response to *M. hiemalis* infection at later time points revealed a significant upregulation of two IPR genes: *pals-5* and *eol-1*. IPR genes are categorized based on their dependence on the transcription factor ZIP-1, and multiple pathways can activate the IPR to induce *pals-5p::GFP* (48). While intracellular infection by Orsay virus and microsporidia, proteasome inhibition, or prolonged heat stress are known inducers of the *C. elegans* IPR, it is possible that *M. hiemalis* could activate *pals-5* through a distinct pathway (48). We determined that *M. hiemalis* caused nematode death by extracellular intestinal perforation. The cellular damage caused by *M. hiemalis* infection may activate the IPR through proteotoxic stress. Alternatively, *M. hiemalis* may activate the IPR through a novel, distinct pathway that recognizes either toxins secreted by *M. hiemalis* or the damage that *M. hiemalis* causes. Future studies could investigate the ZIP-1 dependence of *pals-5::GFP* activation by *M. hiemalis*, as well as a broader survey of IPR gene expression map the specific pathway in which *M. hiemalis* induces the IPR.

Humans mount both innate and adaptive immune responses to invasive Mucorales infections. These fungi often establish infection by evading the initial defenses of host epithelial tissues by entry through traumatic wounds or via ingestion (49, 50). The specific growth and developmental stage of Mucorales also influences the host immune effectors involved. For instance, macrophages and neutrophils detect Mucorales growth following spore detection in the host epithelium (51). Macrophages can inhibit spore germination into hyphae and other growth structures, but they are unable to eliminate resting spores (51). Natural killer cells also play a regulatory role in the human immune response to Mucorales. Cytokines like IL-12 and type-1 interferons activate natural killer cells, and they co-localize with macrophages at infection sites and areas of tissue damage (51). These innate immune effectors play a crucial role in initiating the adaptive immune response in humans, where T cells provide defense against the fungal pathogen through the secretion of antifungal cytokines and defensins (51). *C. elegans* lacks an adaptive immune system and circulating immune cells such as macrophages and neutrophils; hence, its detection of *M. hiemalis* may rely on unique PAMP or DAMP-like receptors capable of recognizing toxins or damage associated with *M. hiemalis* infection. Notably, the *C. elegans* IPR shares similarities with mammalian type-I interferon responses (52). Although the mechanism is unclear, components of the IPR might secrete signaling proteins to activate a two-step response to infection similar to the type-1 interferon pathway (52). These parallels between conserved pathways present an exciting opportunity to characterize a novel *C. elegans* immune response to *M. hiemalis*.

### Conclusion

In conclusion, this study identified the human-pathogenic dimorphic fungus *M. hiemalis* as a novel, naturally occurring pathogen of *Caenorhabditis* nematodes, establishing *Caenorhabditis* as a model for dissecting this host-pathogen interaction. We characterized the fungal life cycle inside nematode hosts, assayed the reduction of nematode lifespan across diverse species, and investigated the host’s potential immune responses to infection. Our findings demonstrate that a wide range of species ingest *M. hiemalis* sporangiospores, and subsequent fungal growth kills nematodes by perforating the intestinal barrier. Our investigation of known *C. elegans* immune pathways revealed a surprising upregulation of two IPR genes, *pals-5* and *eol-*1, which characterizes a novel immune response to *M. hiemalis* infection. This research offers novel insights into the interactions between *Caenorhabditis* nematodes and *M*. hiemalis, which expands the collection of natural nematode pathogens and opens new avenues for research on host-fungus interactions.

## MATERIALS AND METHODS

### Co-culture infection transmission experiment

Wild isolate SOW21 *C. briggsae* and SOW6 *C. elegans* were both chunked onto one Nematode Growth Media (NGM) plate with a flame-sterilized spatula. Co-culture plates were maintained at 20°C and checked daily for reporter fluorescence compared to SOW6-only controls. Positive co-culture tests were imaged on an Olympus SZX10 stereo microscope at 6× magnification. Images were captured with an Olympus DP23 camera on Olympus cellSENS imaging software (https://evidentscientific.com/en/).

### Sequencing of *M. hiemalis* and *C. briggsae*

*M. hiemalis* DNA was isolated with a Quick-DNA Miniprep Kit (Zymo Research) according to the manufacturer’s instructions. PCR amplification of the internal transcribed spacer region was performed with primers described by White et al. (53) (ITS1 – 5′-TCCGTAGGTGAACCTGCGG, ITS4 5’- TCCTCCGCTTATTGATATGC). Sanger sequencing was performed by Azenta (South Plainfield, NJ). The resulting sequence of the *M. hiemalis* JNSP1 isolate was submitted to the NCBI (GenBank: PV719612). ITS sequences of other Mucor species were obtained from the NCBI BLASTN. The phylogeny was inferred using the maximum likelihood method and the Hasegawa-Kishino-Yano model of nucleotide substitutions. The evolutionary rate differences among sites were modeled using a discrete Gamma distribution across four categories (*+G*, parameter = 0.5606). The analytical procedure encompassed 22 nucleotide sequences with 746 positions in the final data set. Evolutionary analyses were conducted in MEGA12 (https://www.megasoftware.net/).

*C. briggsae* (SOW21) DNA was extracted with Quick-DNA Miniprep Kit (Zymo Research). PCR amplification of the 18S ribosomal gene was performed with primers described by Blaxter et al. (54) (SSU18A – AAAGATTAAGCCATGCATG, SSU26R – CATTCTTGGCAAATGCTTTCG). Sanger sequencing was performed by Azenta (South Plainfield, NJ). The results were further confirmed with ITS2 sequencing by the CaeNDR project (https://caendr.org/).

### Maintenance of nematodes

*Caenorhabditis* nematodes and *P. pacificus* were grown and maintained on nematode growth medium (NGM) plates and incubated at 20°C. NGM plates were seeded with *E. coli* OP50-1 bacteria. All strains used in this study and their sources are listed in Table 1.

### Maintenance of *M. hiemalis*

*M. hiemalis* fungus was grown and maintained on Yeast Peptone Glucose (YPG) plates. YPG plates were inoculated by transferring sporangiospores with a sterile pipette tip in an “X” pattern. Plates were incubated at room temperature in the presence of light to encourage sporangiospore production.

### Sporangiospore harvesting and nematode infection

YPG plates were inoculated with *Mucor hiemalis* and grown for 4 days before sporangiospore harvesting; 10 mL sterile ddH2O was pipetted onto YPG plates. A sterile cell spreader was used to scrape the top of the mycelial mat to release sporangiospores. Plates were tilted to one side, and a pipette was used to transfer the spore suspension to a 15 mL conical tube. Sporangiospore concentrations were determined with a hemocytometer. Chlamydospores were not observed during the sporangiospore prep of *M. hiemalis*. Spore solutions were pelleted by centrifugation at 4,300 × *g* for 10 min, and the supernatant was discarded. Spore pellets were resuspended with OP50-1 *E. coli* to a dose of 10 million sporangiospores per 500 μL. NGM plates were seeded with the sporangiospore and OP50-1 mixture and dried in a laminar flow hood. Synchronized nematodes were transferred to fungal plates after plate drying.

### Direct yellow 96 staining

Infected nematodes were washed off plates into microcentrifuge tubes with M9 + 0.1% Tween-20. Nematodes were washed 2× with M9 + 0.1% Tween-20, and the supernatant was discarded. Nematodes were fixed with 700 μL acetone, were incubated overnight, or were stored at 4°C. Samples were washed with 1 mL 1× PBS + 0.1% Tween-20; 500 μL DY96 solution (20 µg/µL DY96, 0.1% SDS in 1× PBS + 0.1% Tween-20) was added, and the samples were incubated for 30 min at 20°C with rotation. Samples were spun down, and the excess DY96 solution was discarded. Vectashield (Vector Laboratories) mounting medium was added to the samples before mounting on glass slides.

### Fluorescence microscopy

Microscopy was performed on an Olympus BX61 epifluorescence microscope using 10× and 40× objectives. Images were captured using an Olympus DP21 (Fig. 2) or a Tucsen Dhyana 400D (Fig. 5) camera. µManager (https://micro-manager.org/) software was used to take Z-stacks of nematodes. ImageJ FIJI (https://fiji.sc/) was used to analyze fluorescence images.

### Propidium iodide and direct yellow 96 live staining

In total, 100 N2 *C. elegans* were synchronized by bleaching and grown to adult day 1 stage. Standardized *M. hiemalis* sporangiospore doses were added to each infection plate, and nematodes were incubated at 20°C. Nematodes were collected for analysis at 1–4 days after infection. Nematodes were washed off the plates into microcentrifuge tubes with M9 + 0.1% Tween-20 and washed 2× with M9; 500 µL DY96 live staining solution (20 µg/µL DY96, 1× PBS + 0.1% Tween-20) and 30 μL propidium iodide (Thermo Scientific) was added. Samples were incubated for 1 h at 20°C with rotation. Samples were spun down, and the excess dye was discarded. Samples were washed 2× with PBST. Vectashield (Vector Laboratories) mounting medium was added to samples before mounting on slides. Image J FIJI was used to view image stacks. Nematodes were scored positive for intestinal perforation if cells outside of the intestinal lumen were stained with PI. 20 nematodes were scored per trial, and the experiment was repeated three times independently.

### Survival assay

Survival assays to determine the host range of *M. hiemalis* were performed with the following species: *C. elegans* (N2), *C. japonica* (DF5081), *C. tropicalis* (JU1373)*, C. briggsae* (SOW21), and *P. pacificus* (PS312). Survival assays were carried out in biological triplicates with spore treatments and controls. Nematodes were synchronized by bleaching and grown to larval stage 4; 20 nematodes were transferred to either infection plates with sporangiospore + OP50-1 mixture or control plates seeded with OP50-1 and incubated for 2 days. Nematodes were tested for survival every day by prodding with a platinum wire. Nematodes were transferred to new plates every 2 days to prevent scoring interference by progeny. Statistical analysis of nematode survival was performed with log-rank (Mantel-Cox) analyses in Graphpad Prism v10 software.

### Food preference assay

Square Petri dishes with grids were prepared with NGM media. Preference assay plates were inoculated with sporangiospores or sporangiospore mixture with OP50-1, as shown in Fig. 3. Spore conditions were at a dose of 1 million sporangiospores in either 200 µL sterile ddH2O or OP50-1. Plates were dried in a laminar flow hood, and 100 µL sodium azide (0.5M) was added to each choice to prevent travel between choices. About 100 synchronized adult day 1 N2 *C. elegans* or *C. briggsae* were placed in the middle four squares on the choice plate. A Kimwipe was used to remove excess M9 buffer. Nematodes were counted after 1 h and were scored based on location and choice. Experiments were carried out in biological triplicate. Preference indexes per trial were calculated and averaged. Statistical analysis of nematode choice preference was performed in Prism software. Means were analyzed with a one-sample *t*-test against a theoretical mean of zero.

### Late-stage (days 3 and 5 post-infection) immune response quantification of *C. elegans* by qRT-PCR

In total, 1,000 N2 *C. elegans* were synchronized and grown to the adult day 1 stage in biological triplicates. *M. hiemalis* infection plates were inoculated with 10 million sporangiospores per 500 μL of OP50 as previously described. Orsay virus infection plates were inoculated with 200 µL Orsay virus filtrate to serve as a positive control of IPR gene expression. Control plates were inoculated with 500 μL OP50-1. Orsay virus infection plates were inoculated with 200 µL Orsay virus filtrate to serve as a positive control of IPR gene expression; 500 μL of *E. coli* expressing Exotoxin A (ToxA) from *P. aeruginosa* (55) was inoculated onto separate infection plates to serve as a positive infection control for *irg-1* gene expression; 500 μL of control *E. coli* without the promoter for ToxA expression was inoculated onto separate plates as a control for the ToxA expression. Nematodes were collected for RNA extraction at 3 and 5 days post-infection. Nematodes were washed off plates with M9 buffer into 15 mL conical tubes and subjected to gravity flotation 5× to remove excess progeny. Nematodes were concentrated and transferred to microcentrifuge tubes. RNA was extracted using TRI-zol by the Direct-zol RNA kit (Zymo Research). cDNA was made from extracted RNA with the Maxima H Minus First Strand cDNA Synthesis Kit (Thermo Scientific). qRT-PCR was performed using Brilliant III Ultra-Fast SYBR Green qPCR Master Mix (Agilent) on a Stratagene Mx3005P QPCR instrument. All qRT-PCR primers used are listed in Table 2. Each qRT-PCR was measured in technical duplicate. Gene expression data were normalized to *snb-1* gene expression. The ΔΔCt method was used to calculate gene expression fold changes, and analysis was performed in GraphPad Prism v10. One-sample *t*-tests were performed to compare gene expression fold changes to a theoretical mean of 1.

**TABLE 2:** qRT-PCR primers used in this study^*a*^

Gene	Primer sequences	Source
*snb-1*	F - CCGGATAAGACCATCTTGACG R - GACGACTTCATCAACCTGAGC	(18)
*pals-5*	F - CATTGGAAAGCGATATTGGA R - TCTCCAGGCACCTATCTTGTAG	(18)
*eol-1*	F - GAAGGAGGTGGCGATGTTTAT R - CGGCGTCGATTGTCTCTTT	(18)
*skr-5*	F - CGAAGAGCAAGATGTCAAAATTG R - AGAAGCTTGGATTGATTGGCA	(18)
*skr-4*	F - CCGACAGCCAGAAACAAATCA R - GGTCTTGGATTGGCTGATCAC	(18)
*cul-6*	F - CTGGGCTTACTCACAATGCC R- GCAGAGTTGGCTTGCTGTAA	(48)
*lys-1*	F - TGCTTTGGCTTCTGCTAGACCACA R - CACAACTTCCGGAGCTGGCTCG	Designed for this study
*daf-7*	F - TCAGCATTCAACTTCCAATTGATAC R- CGGAGAAATTGTGAACCAACTTT	(56)
*irg-1*	F - TGTGGAGGCCTCACCAACCGAT R - ACACCGCTGGTCTGCTTTGTCA	Designed for this study

^*a*^ Sequences and sources for all qRT-PCR primers used in this study.

### Immune response quantification of larval 4 and adult day 2 *C. elegans* by qRT-PCR

In total, 1,000 N2 *C. elegans* were synchronized and grown in biological triplicates. Infection plates were inoculated with 10 million sporangiospores per 500 μL of OP50 as described previously at either larval stage 4 or adult day 2 stages (Fig. S2). Control plates were inoculated with 500 μL OP50-1. Nematodes were incubated at room temperature for 2 days after infection before collection for RNA extraction. Collection of nematodes, RNA extraction, cDNA synthesis, qRT-PCR, and analysis were performed as described above.

## SUPPLEMENTAL MATERIAL

Fig. S1 iai.00310-25-s0001.pdf Accumulation and germination of fungi within the worm intestine.

Fig. S2 iai.00310-25-s0002.pdf Additional gene expression data.

ASM does not own the copyrights to Supplemental Material that may be linked to, or accessed through, an article. The authors have granted ASM a non-exclusive, world-wide license to publish the Supplemental Material files. Please contact the corresponding author directly for reuse.
