# Supplementary material for: Novel infection by Mucor hiemalis kills Caenorhabditis hosts through intestinal perforation
Source: Infect Immun. 2026 Apr 15;94(5):e00310-25. doi: 10.1128/iai.00310-25 (PMC13163196; doi:10.1128/iai.00310-25)
Supplement: Fig. S1 — Accumulation and germination of fungi within the worm intestine. [file iai.00310-25-s0001.pdf]

A

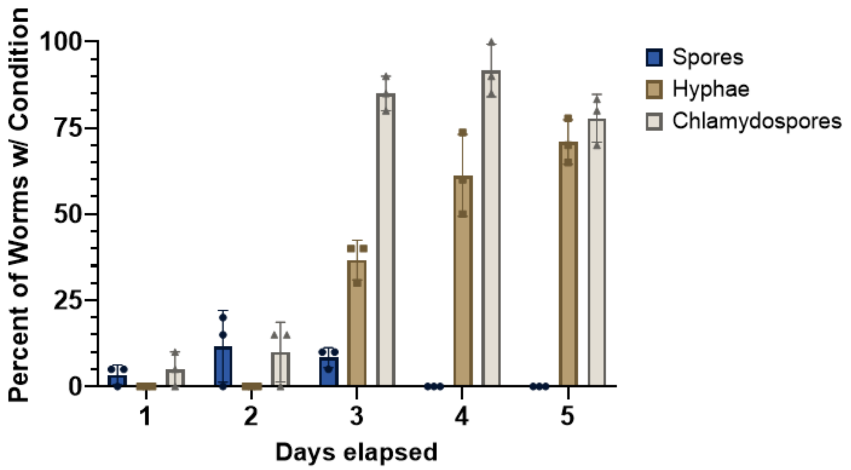


B

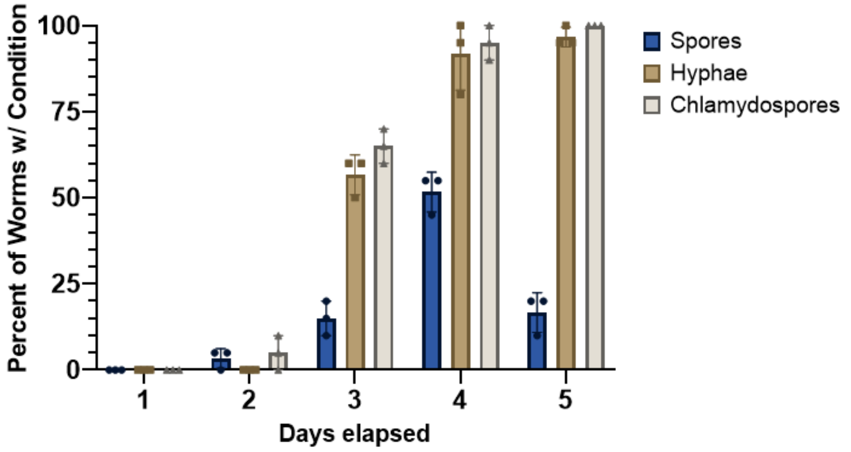


**Figure S1: Ingested *M. hiemalis* sporangiospores germinate hyphal bodies and accumulate inside of the *Caenorhabditis* nematode intestine.**

Data from fluorescence micrographs of *M. hiemalis* growth stages stained with DY96 in infected wild type N2 *C. elegans*. *C. elegans* were scored for fungal growth conditions in biological triplicates. Graphs show the percentage of nematodes with *M. hiemalis* sporangiospores, hyphae, or chlamydospores. *M. hiemalis* growth stage counts in N2 *C. elegans* across 5 days of observation by fluorescence microscopy with (A) adult day 2 (B) adult day 3 nematodes. Experiment was repeated 3 times independently (n = 20 for each experiment). Error bars show standard deviation.
