# Supplementary material for: Novel infection by Mucor hiemalis kills Caenorhabditis hosts through intestinal perforation
Source: Infect Immun. 2026 Apr 15;94(5):e00310-25. doi: 10.1128/iai.00310-25 (PMC13163196; doi:10.1128/iai.00310-25)
Supplement: Fig. S2 — Additional gene expression data. [file iai.00310-25-s0002.pdf]

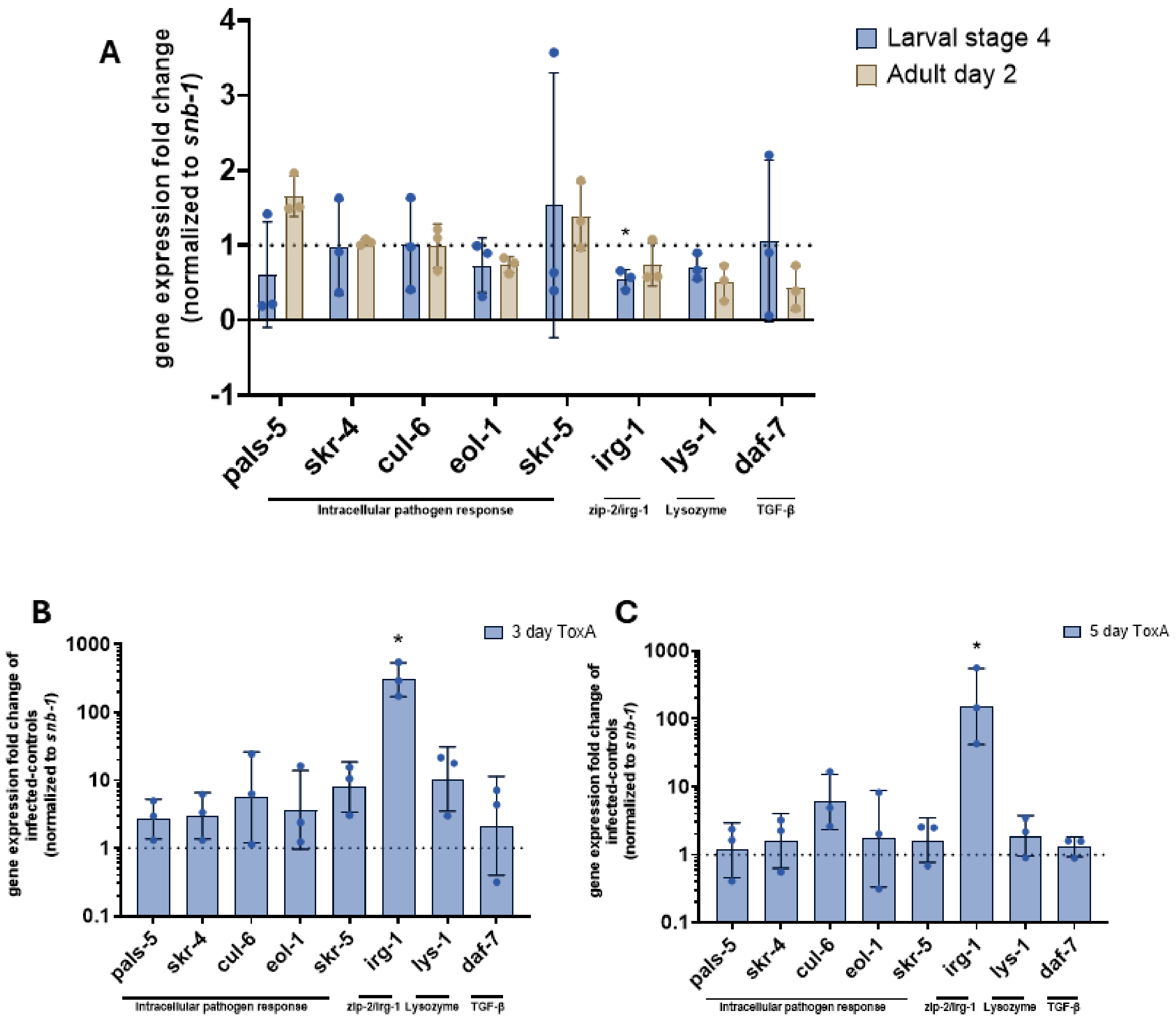

Figure S2: IPR and other immunity genes are not upregulated in response to *M. hiemalis* infection. A) 1000 synchronized larval stage 4 and adult day 2 N2 *C. elegans* were incubated on fungal infection plates and OP50-1 control plates for 2 days before RNA extraction. Experiment was repeated 3 times independently. Relative fold changes in gene expression were normalized to *snb-1* and compared to control groups. Graph shows gene expression comparison between larval stage 4 and adult day 2 nematodes infected with *M. hiemalis*. Error bars show standard deviation. Significance was determined by comparing gene expression fold changes of infected vs. controls to a value of 1 with one-sample t-tests. IPR genes *skr-4, skr-5, cul-6, pals-5,* and *eol-1* are not induced during *M. hiemalis* infection in larval stage 4 or adult day 2 *C. elegans*. Innate immunity genes *daf-7* and *lys-1* are also not induced. *irg-1* gene expression is reduced upon *M. hiemalis* infection in larval stage 4 nematodes ($p = 0.0264$, $n = 3$). B) Gene expression fold change of seven IPR and innate immunity genes of *C. elegans* cultured with ToxA secreting *E. coli* after 3 days. Error bars show standard deviation. Significance was determined by comparing gene expression fold changes of nematodes cultured with ToxA vs. controls to a value of 1 with one-sample t-tests. *irg-1* ($p = 0.0033$) is significantly upregulated in nematodes cultured with ToxA secreting *E. coli*. C) Gene expression fold change of IPR and innate immunity genes of *C. elegans* cultured with ToxA secreting *E. coli* after 5 days. Error bars show standard deviation. Significance was determined by comparing gene expression fold changes of nematodes cultured with ToxA vs. controls to a value of 1 with one-sample t-tests. *irg-1* ($p = 0.0211$) is significantly upregulated in nematodes cultured with ToxA secreting *E. coli*.
